# Supplementary material for: Transition between distinct hybrid skyrmion textures through their hexagonal-to-square crystal transformation in a polar magnet
Source: Nat Commun. 2023 Dec 5;14:8050. doi: 10.1038/s41467-023-43814-x (PMC10698042; doi:10.1038/s41467-023-43814-x)
Supplement: Supplementary file 1 — Supplementary Information [file 41467_2023_43814_MOESM1_ESM.pdf]

Supplementary Information:

Transition between distinct hybrid skyrmion textures through their hexagonal-to-square crystal transformation in a polar magnet

Deepak Singh[1*], Yukako Fujishiro[2], Satoru Hayami[3], Samuel H. Moody[1], Takuya Nomoto[4], Priya R. Baral[1], Victor Ukleev[5], Robert Cubitt[6], Nina-Juliane Steinke[6], Dariusz J. Gawryluk[7], Ekaterina Pomjakushina[7], Yoshichika Ōnuki[2], Ryotaro Arita[4,2], Yoshinori Tokura[8,2], Naoya Kanazawa[9], and Jonathan S. White[1*]

[1]*Laboratory for Neutron Scattering and Imaging (LNS), Paul Scherrer Institute (PSI), CH-5232 Villigen, Switzerland*

[2]*RIKEN Center for Emergent Matter Science (CEMS), Wako, Saitama 351-0198, Japan*

[3]*Graduate School of Science, Hokkaido University, Sapporo 060-0810, Japan*

[4]*Research Center for Advanced Science and Technology, University of Tokyo, Komaba, Meguro-ku, Tokyo 153-8904, Japan*

[5] *Helmholtz-Zentrum Berlin für Materialien und Energie, D-14109 Berlin, Germany*

[6] *Institut-Laue-Langevin, 6 rue Jules Horowitz, Grenoble 38000, France*

[7] *Laboratory for Multiscale Materials Experiments (LMX), Paul Scherrer Institut (PSI), CH-5232 Villigen PSI, Switzerland*

[8] *Department of Applied Physics, The University of Tokyo, Bunkyo, Tokyo 113-8656, Japan*

[9] *Institute of Industrial Science, The University of Tokyo, Meguro-ku, Tokyo 153-8505, Japan*

* To whom correspondence should be addressed. E-mail: deepak.singh@psi.ch, jonathan.white@psi.ch

Supplementary Note I : Phase diagram of $EuNiGe_3$ from ac susceptibility measurements¨
To complement the data shown in Fig. 2b, we performed similar measurements between 2K and 14.5K. Consistent with the scattering experiments, the majority of the susceptibility data were collected as a function of increasing magnetic field. The phase contour map was constructed from our χ_{ac}' data and is shown in Supplementary Fig. 1a. At lower temperatures, the transition between phases I and II is marked by a step-like increase in the χ_{ac}'. While close to T_N, the same transition is hallmarked by the appearance of a sharp peak. Making connection with our SANS data, this peak in the susceptibility curve is reminiscent of a topologically non-trivial transition between a multi-domain single-Q spiral order and a multi-Q skyrmion lattice. As shown in Supplementary Fig. 1b, the transitions into and out of phases II and III are also accompanied by discontinuous changes in χ_{ac}''. These observations are in-line with the interpretation that these phase transitions involve various energetically stable multi-Q skyrmion phases.

Supplementary Note II : Analysis of ρ_{yx} data
We compare the magnetic phase diagram determined by the magnetisation, $M(H,T)$, and SANS data for H || [001] in $EuNiGe_3$ with the the topological response of each phase probed by the topological Hall resistivity ρ_{yx} (Supplementary Fig. 2a). The transverse resistivity ρ_{yx}, is generally made up of three components:

$$\rho_{yx} = R_0 B + S_A \rho_{xx} + \rho_{yx}^{THE} \quad (1)$$

where the first and second terms are the normal and anomalous Hall resistivities proportional to the magnetic induction field B and the magnetisation M, respectively, and the third term represents the topological component. To determine the topological Hall effect (THE) contribution ρ_{yx}^{THE}, the normal and the conventional anomalous contributions is subtracted from the measured ρ_{yx}. As shown in Supplementary Fig. 2b, the magnitude of THE signal at T = 2 K is between ~0.01-0.02 $\mu\Omega$.cm in Phase II and III regions, which indicates that the application of H induces topological phase transitions in the context of spin textures. It is important to note that we also observe unexpected finite topological Hall signal in phase I and Phase IV from the fitting. However, we did not see any hysteretic component in the angle-dependent ρ_{yx} measurements for Phase I and IV (see Fig. 2d), which suggest that the THE signal in Supplementary Fig. 2b (for phase I and IV) is unlikely to be due to the presence of topological magnetism.

There could be different reasons for the unexpected finite ρ_{yx} in phases I and IV. For example, Equation 1, which is used to calculate ρ_{yx}^{THE}, includes the assumption that the normal Hall coefficient R_0, which is also included in the topological Hall coefficient, is unchanged across different magnetic phases. However, this hypothesis is only valid if the helical structure has a long period. In other words, in the presence of weak DMI, the magnetic order cannot modify the electronic structure dramatically, and the normal Hall coefficient, R_0, is not affected by variation in the magnetic order. Since the itinerant electrons are undoubtedly coupled to the magnetism in this system, we thus conclude pronounced changes likely take place in the electronic structure between the different phases. As a result, we can expect R_0 to have a H-dependency between the ground state phase I and the saturated regime. Therefore, the standard analysis of the topological Hall signal based on a H-independent R_0 cannot apply here. This likely leads to the formation of an uncompensated topological signal in phase I and IV.

On the other hand, if the conventional analysis approach for ρ_{yx} data would in fact be appropriate, the additional Hall signal observed in phases I and IV may then have its origin in an extrinsic mechanism. A possible explanation is skew scattering from topologically non-trivial features of domain walls separating different magnetic domains [S1-S3]. Since phases I and IV are determined to naturally form multi-domain states according to our SANS measurements, topological defects may be expected to exist. Thus, the magnitude of skew scattering-related Hall resistivity may exceed the intrinsic topological Hall effect. Furthermore, such scattering events are less likely to occur in the single-domain multi-Q phases II and III

Supplementary Note III : Spin Polarisation Factor

Here we attempt a quantitative analysis of the THE, whereby we assume the magnetic order in phases II and III correspond to skyrmion phases. According to the continuum approximation, the emergent magnetic field due to a skyrmion is given by $B_{\mathrm{em}} = -\Phi_0/\lambda_{Sk}^2 = -(h/e)\, n_{Sk}$, where λ_{Sk} represents the magnetic period of the skyrmion lattice (SkL), and n_{Sk} is the skyrmion density. From Fig. 3d, we obtain $n_{Sk} \sim 0.22$ nm^{-2} in Phase II and $n_{Sk} \sim 0.184$ nm^{-2} in Phase III, which yields $B_{\mathrm{em}} \sim -900$ T and -750 T, respectively. Such enormous effective fields can be related empirically to the topological Hall resistivity through the normal Hall coefficient R_0, and the effective spin polarisation P of conduction electrons given by [S4,S5]:

$$\rho_{Sk}^{THE} = P.R_0.B_{em} \qquad (2)$$

After roughly extracting topological Hall resistivity $\rho_{Sk}^{THE} \sim 0.02\ \mu\Omega$.cm and $R_0 \sim 0.03\ \mu\Omega$.cm/T from Fig. 2c for both Phases II and III, we estimate $P = 7 \times 10^{-4}$ and 8×10^{-4}, respectively.

In general, *P* is relatively strong for *d*-*d* coupling in transition metal compounds (e.g. the *B*20 family). For example $P \simeq 0.1$ for MnSi [S4], $P \sim 0.25$-0.38 for MnSi under pressure [S5], and for slightly-doped $Mn_{1-x}Fe_xSi$, $P \sim 0.3$-0.45 [S6]. However, it is expected to be small for rare earth systems with moderate *f*-*d* coupling, for example $P = 0.07$ for Gd_2PdSi_3 [29]. We performed a spin density functional theory (SDFT) calculation of the density of states (DOS) near the Fermi level of $EuNiGe_3$, and estimate a small value of $P \sim 0.015$. The small estimated size of *P* can be attributed to the dominant contribution of Ni and Ge to the DOS compared with Eu. While the estimated value for *P* from SDFT is larger than those estimated above from the experimental data, the small sizes of both theoretical and experimental *P* estimates prevent a meaningful quantitative comparison. Nonetheless, the SDFT result suggests the small contribution of Eu electron bands at the Fermi level, and is thus generally consistent with a small value of *P*, and consequently the small topological Hall resistivity observed in this compound.

Supplementary Note IV : Experimental data for H parallel [100]

In Supplementary Fig. 3 we show experimental data obtained for progressively stronger magnetic fields applied along the [100] direction, i.e. perpendicular to the polar axis. Supplementary Fig. 3a shows the *H*-*T* phase diagram constructed from temperature and *H*-dependent magnetic susceptibility measurements. Below saturation, the constructed phase diagram displays two broad regions, with the low field, low temperature portion demarcated by a kink in the susceptibility curves (data not shown).

Unpolarised SANS data obtained for *H* || [100] were measured at the SANS-I beamline, PSI, and are shown in Supplementary Figs. 3b and c. In the chosen experimental setup with *H* perpendicular to the neutron beam, we could observe only two of the expected single-*Q* domains described by $\mathbf{Q_1}$ in phase I, namely those with *Q*-vectors aligned closest to both *H* and the horizontal axis. The other two domains that would be expected to show scattering in the top and bottom portions of the detector were shadowed by the more restricted neutron beam access of the horizontal field magnet compared with the *H* parallel to beam geometry used for the *H* || [001] study discussed in the main text. Therefore, as seen in Supplementary Fig. 3b, just two single-*Q* domains are observed after zero-field cooling, and in addition with different relative intensities presumable related to the different populations of single-*Q* domains in the crystal.

Turning the *H*-dependence of the observable magnetic scattering at 1.9 K, as seen in Supplementary Fig. 3b we do not observe the magnetic *Q*-vectors to display a drastic rearrangement up to saturation. Instead, a smooth variation of the overall scattering intensity is

observed as the field is increased (Supplementary Fig. 3c), with a small increase seen in the low field region. Notably there is no obvious feature in the high field portion related to the phase line determined by susceptibility shown in Supplementary Fig. 3a. Therefore, further work is needed to clarify the physical origin of this feature. The variation in SANS intensity in the low field region may have its origin in a differing behaviour of the unobserved single *Q*-domains which have *Q*-vectors nearly perpendicular to *H*, or the deformation of the observed ground state into a conical-like structure. Again, further experiments are needed to elucidate the fate of all single-*Q* domains under in-plane fields. Thus, here we focus on the behaviour of the two observable single-*Q* domains. Since they both survive until saturation, this provides evidence that the modulation of the ground state phase I has a mainly helical character, since the stability of helical structures benefit from the generally enhanced susceptibility for fields applied at angles to the helical plane. This contrasts with the expectation for a cycloidal modulation with moments rotating in the plane containing *Q*, and for which the susceptibility is enhanced normal to the cycloidal plane. In the cycloidal modulation scenario, the observed single-*Q* domains could be expected to be destabilised (or polarised) well before saturation, and instead it would be the unobserved single-*Q* domains that would be more likely to survive until saturation. Therefore, the data at hand provide support that the ground state phase I in $EuNiGe_3$ has a significant helical character.

Supplementary Note V : Field tilted SANS measurements

In the field-tilted SANS measurements performed in Phase II we studied the effect on the hexagonally coordinated multi-*Q* domains when the magnetic field of 2.6 T was applied at increasing angle to the polar axis within the (110) plane. Experimentally, the sample was always zero-field cooled (ZFC), before the field applied at progressively larger tilt angles up to 15° from the *c*-axis. Due to the mutually orthogonal alignment of the two domains for $H \parallel$ [001], the tilting of the field away from *c* in the (110) plane naturally breaks the symmetry for the multi-*Q* domain formation, and is thus is expected to affect the stability of the two domains differently.

The experimental configuration for these unpolarised SANS measurements is illustrated in Supplementary Fig. 4a, with the magnetic field tilt angle away from the [001] axis labelled as ø. For ø = 0°, Supplementary Fig. 4b shows we observe the 12-spot pattern due to the two-domain triple-*Q* state, where all *Q*-vectors are the same as described in Fig. 3j from the main text. For ø = 15° however, Supplementary Fig. 4b shows that indeed one of the hexagonal multi-*Q* domains is indeed preferentially stabilised compared with the intensity due to other domain severely suppressed. The ø -dependence of the overall SANS intensity due to each of the two domains is shown in Supplementary Fig. 4d. The inverse correlation between the ø -

dependence of the SANS intensities due the two domains confirms their triple-Q composition, and the allocation of the original state at 2.6 T and 1.9 K for H || [001] as being due to a coexistence of two triple-Q domains.

Supplementary Note VI : Distortion of the hexagonal skyrmion crystal Phase II

In Phase II the constituent Q-vectors in each of the two multi-Q domains do not describe perfectly hexagonal skyrmion crystals. Instead the configuration of Q-vectors describes distorted hexagonal skyrmion crystals. Since the reciprocal skyrmion lattice can be generally described in terms of a 2D Bravais lattice, the observed hexagonal lattice distortion implies directly that in real-space the skyrmions display an elliptical, and not circular, radial cross section. As an example, Supplementary Fig. 6a shows the SANS data obtained from Phase II at 2.6 T and 2 K. The distribution of diffraction peaks from one of the skyrmion domains is indeed shown to be elliptical, as shown by the overlaid ellipse. Using the relation $\varepsilon = 1/(\sqrt{3}\tan(\theta/2))$, where θ is the angle between $\mathbf{Q}_2$ and - $\mathbf{Q}_4$ (the two directions indicated in Supplementary Fig. 6a), we parameterise the ellipticity ε of the skyrmion distortion directly. The H-dependence of ε in Phase II determined from our SANS data is shown in Supplementary Fig. 6b. Since $\varepsilon = 1$ arises for $\theta = 60°$, values of $\varepsilon > 1$ like those we observe imply θ values less than 60°. In detail, we find θ to display a small H-dependent variation within Phase II from ~55.5° to ~53.5° as ε varies from ~1.10 to ~1.15. The effect in real-space is thus a distortion of the skyrmions along the direction of $\mathbf{Q}_3$.

Supplementary Note VII : Spin Model Parameters

We present the model parameters used in the numerical simulations. The results in Fig. 6 are obtained by setting

$$J_{\mathbf{Q}_1} = J_{\mathbf{Q}_2}/\kappa_1 = J_{\mathbf{Q}_3}/\kappa_1 = J_{\mathbf{Q}_4}/\kappa_1 = J_{\mathbf{Q}_5}/\kappa_2 = J_{\mathbf{Q}_6}/\kappa_2 \quad (3)$$

for the exchange interactions, and

$$|\mathbf{D}_{\mathbf{Q}_1}| = |\mathbf{D}_{\mathbf{Q}_5}|/\kappa_2 = |\mathbf{D}_{\mathbf{Q}_6}|/\kappa_2 \equiv D \quad (4)$$

$$|\mathbf{D}_{\mathbf{Q}_3}| \equiv D' \quad (5)$$

$$|\mathbf{D}_{\mathbf{Q}_2}| = |\mathbf{D}_{\mathbf{Q}_4}| = 0 \quad (6)$$

for the DMIs. Moreover, the DMIs at each ordering wavevector are decomposed as

$$\mathbf{D}_{\mathbf{Q}_v} = \mathbf{D}^p_{\mathbf{Q}_v} + \mathbf{D}^r_{\mathbf{Q}_v} \quad (7)$$

where $\mathbf{D}^p_{\mathbf{Q}_\nu}$ and $\mathbf{D}^r_{\mathbf{Q}_\nu}$ stand for the polar- and radial-type DMIs; $\mathbf{D}^p_{\mathbf{Q}_\nu} \perp \mathbf{Q}_\nu$ and $\mathbf{D}^r_{\mathbf{Q}_\nu} \,||\, \mathbf{Q}_\nu$. We set

$$\mathbf{D}^p_{\mathbf{Q}_1} = \frac{D^p_1}{|\mathbf{Q}_1|}\left(-Q^y_1, Q^x_1\right) \tag{8}$$

$$\mathbf{D}^r_{\mathbf{Q}_1} = \frac{D^r_1}{|\mathbf{Q}_1|}\left(-Q^x_1, -Q^y_1\right) \tag{9}$$

$$\mathbf{D}^p_{\mathbf{Q}_3} = \frac{1}{|\mathbf{Q}_3|}\left(-Q^y_3, Q^x_3\right) \tag{10}$$

$$\mathbf{D}^r_{\mathbf{Q}_3} = 0 \tag{11}$$

$$\mathbf{D}^p_{\mathbf{Q}_5} = \frac{1}{|\mathbf{Q}_5|}\left(-Q^y_5, Q^x_5\right) \tag{12}$$

$$\mathbf{D}^r_{\mathbf{Q}_5} = 0 \tag{13}$$

$$\mathbf{D}^p_{\mathbf{Q}_6} = \frac{1}{|\mathbf{Q}_6|}\left(-Q^y_6, Q^x_6\right) \tag{14}$$

$$\mathbf{D}^r_{\mathbf{Q}_6} = 0 \tag{15}$$

with D^p_1 = 0.9 and $D^r_1 = \sqrt{1-\left(D^p_1\right)^2}$. Finally, we set $\left(\alpha_{\mathbf{Q}_\nu}, \beta_{\mathbf{Q}_\nu}, \gamma_{\mathbf{Q}_\nu}\right)$ as follows:

$$\alpha_{\mathbf{Q}_1} = \alpha_{\mathbf{Q}_5} = \alpha_{\mathbf{Q}_6} \equiv \alpha \tag{16}$$

$$\alpha_{\mathbf{Q}_2} = \alpha_{\mathbf{Q}_3} = \alpha_{\mathbf{Q}_4} \equiv \alpha' \tag{17}$$

$$\beta_{\mathbf{Q}_1} = \beta_{\mathbf{Q}_3} = \beta_{\mathbf{Q}_5} = \beta_{\mathbf{Q}_6} = 0 \tag{18}$$

$$-\beta_{\mathbf{Q}_2} = \beta_{\mathbf{Q}_4} \equiv \beta \tag{19}$$

$$\gamma_{\mathbf{Q}_1} = \gamma_{\mathbf{Q}_3} = \gamma_{\mathbf{Q}_5} = \gamma_{\mathbf{Q}_6} = 0 \tag{20}$$

$$\gamma_{\mathbf{Q}_2} = \gamma_{\mathbf{Q}_4} \equiv \gamma \tag{21}$$

where α = 1.05, α' = 1.35, β = 0.12 / $\sqrt{5}$, and γ = 0.06 / $\sqrt{5}$. In the calculations, we consider all the ordering wavevectors that are symmetry-equivalent to $\mathbf{Q}_1$-$\mathbf{Q}_6$.

The role of the above model parameters is as follows. κ_1 and κ_2 represent the interaction ratios to the interaction channel at $\mathbf{Q}_1$; the interaction in the $\mathbf{Q}_1$ channel is dominant, while those in $\mathbf{Q}_2$-$\mathbf{Q}_6$ are subdominant. This choice of the parameters leads to the spiral state with $\mathbf{Q}_1$ at zero field. In Eqs. (8)-(15), we set the model parameters in terms of the DMI so that the helicity of the spiral and skyrmion phases observed in experiments is reproduced. In particular, the radial component in Eq. (7) plays an important role in determining the helicity in Phase I and Phase III. Meanwhile, the DMI plays a lesser role in the stabilization and the choice of the helicity

in Phase II, since the DMIs at $\mathbf{Q}_2$-$\mathbf{Q}_4$ hinder the superposition of the triple-Q spiral waves, i.e., the hexagonal skyrmion lattice. Thus, in Phase II, another interaction is necessary to stabilize the skyrmion lattice. This leads us to consider the effect of the symmetric anisotropic exchange interaction in Eqs. (16)-(21), which then plays a role in stabilizing the skyrmion lattice.

Supplementary Note VIII : Spin and Chirality related quantities

We introduce the spin- and chirality related quantities, which are used for the identification of the magnetic phases obtained by the simulated annealing. In the spin sector, we compute the $\mu = x, y, z$ component of the magnetic moment with wave vector **q**, whose expression is given by

$$m_{\mathbf{q}}^{\mu} = \sqrt{\frac{S_s^{\mu}(\mathbf{q})}{N}} \quad (22)$$

$$S_s^{\mu}(\mathbf{q}) = \frac{1}{N}\sum_{j,l} S_j^{\mu} S_l^{\mu} e^{i\mathbf{q}\cdot(\mathbf{r}_j-\mathbf{r}_l)} \quad (23)$$

where $S_s^{\mu}(\mathbf{q})$ is the spin structure factor; $\mathbf{r}_j$ is the position vector at site j. The uniform magnetisation is calculated by $M^{\mu} = (1/N)\sum_i S_i^{\mu}$, which corresponds to the **q** = 0 component of $m_{\mathbf{q}}^{\mu}$.

In the chirality sector, we calculate the spin scalar chirality defined by the triple scalar product of neighboring spins given by

$$\chi_0 = \frac{1}{N}\sum_{\mathbf{R}} \mathbf{S}_j . (\mathbf{S}_k \times \mathbf{S}_l) \quad (24)$$

where **R** denotes the position vectors at the centers of the upward and downward triangles consisting of spins $\mathbf{S}_j$, $\mathbf{S}_k$, and $\mathbf{S}_l$ in counterclockwise order. The nonzero χ_0 indicates the emergence of the topological spin textures, such as the skyrmion lattice.

Supplementary Note IX : Quantification and spatial distribution of helicity (χ) for Néel, Bloch, and hybrid skyrmions

In Figs. 1a, b, e, and f we present schematic representations of different classes of skyrmions considered in this work, namely Néel, Bloch and hybrid skyrmions in Phase II and Phase III of $EuNiGe_3$. Each of these skyrmion types are distinguished by both the magnitude and spatial distribution of helicity (χ) across the skyrmion. In this Supplementary Note, we describe in

detail how the helicity of each skyrmion type is quantified and, in addition, how the spatial distribution of helicity varies amongst the different skyrmion types.

To begin, we elaborate on how the real-space spin textures of Néel, Bloch, and hybrid skyrmions are generated in the absence of genuine atomistic models for magnetic textures in $EuNiGe_3$. The real-space schematics of skyrmions are created by employing a Fourier-space description, which involves a summation over different magnetic components:

$$\boldsymbol{m}(\boldsymbol{r}) = \sum_i \sum_j \boldsymbol{m}_{ij} \cos \boldsymbol{q}_i \cdot \boldsymbol{r} + \phi_{ij}. \tag{25}$$

Here, $\boldsymbol{m}(\boldsymbol{r})$ represents the magnetization vector as a function of spatial coordinates ($\boldsymbol{r}$), where $\boldsymbol{m}_{ij}$ is a parameter describing the magnetization contributed by the ith Fourier component. These components have their orientations and magnitudes determined using a basis scheme indexed by j. Each Fourier component resides at a wavevector $\boldsymbol{q}_i$ and is assigned a phase parameter ϕ_{ij} within the basis. Magnetic skyrmion lattices are generated by constructing a two-dimensional grid of points with varying r. Equation 25 can then be evaluated once the relevant magnetic wavevectors and their corresponding magnetic Fourier components are known.

In our approach, we adopt a scheme in which we set the in-plane magnetic components to have a phase of ϕ_{ij}=0, while the out-of-plane directions are given a 90° phase shift between each wavevector. Through such a scheme, spatial variations in the magnitude of the magnetic moments are minimized, which is in alignment with the expectation of our numerical simulations.

Additionally, provided that a 90° phase difference is maintained between the in-plane and out-of-plane ϕ_{ij} values, a global phase shift, denoted as δ_i, results in a translation of the skyrmion lattice when placed on a rectangular grid. Consequently, the positions of skyrmion cores can be arbitrarily adjusted by varying the value of δ_i. Here, we choose $\delta_i = 0$ such that a skyrmion-core is located at $r = 0$.

Armed with models for the various real-space spin textures, we now move on to quantifying the overall helicity distribution for each type of skyrmion by evaluating χ at over 10000 evenly spaced locations within the magnetic unit cell. The calculated helicity distributions are shown in Supplementary Fig. 7b, d, f, and h, along with the schematic depictions of the various skyrmion types investigated in Supplementary Fig. 7a, c, e, and g. In the insets for Supplementary Figs. 7b, d, f, and h, we report estimates for the mean helicity $\bar{\chi} = \sum_i n_i \chi_i / N$,

and standard deviation $\sigma = \sqrt{\sum_i n_i (\chi_i - \bar{\chi})^2/(N-1)}$, where in each equation n_i is the frequency of the ith helicity bin, χ_i is the mid-point of the helicity bin, and N is the number of locations in the unit cell over which the helicity was calculated.

For the archetypal Néel and Bloch skyrmions, the histograms shown in Supplementary Figs. 7b and d are dominated by a single peak near either $\chi = 0$ or $\pi/2$, as expected for skyrmions with isotropic distributions of helicity. Small contributions to χ slightly away from these peaks are observed, which we attribute to the breakdown of radial symmetry towards the edges of the unit cell and the geometry of the skyrmion lattice (Supplementary Figs. 7a and c). This difference in spatial profile between an azimuthally symmetric skyrmion profile (denoted by either a circle or ellipse for Phase II) and the magnetic unit cell plays an observable role in the χ distribution at the extremities of the latter. The result is minor contributions to the histogram at values deviating slightly beyond the range expected for a single isolated skyrmion in a ferromagnetic background. For the Néel and Bloch type skyrmion histograms, the result is a deviation from a truly singular helicity value, a value of $\bar{\chi}$ slightly different to either 0 or $\pi/2$, and a small, yet finite value for σ.

Nonetheless, when comparing between the calculated χ distributions of the different skyrmion types in Supplementary Figs. 7b, d, f, and h, the histograms for Bloch and Néel skyrmions display a clearly much narrower distribution compared with those of hybrid skyrmion Phases II and III in $EuNiGe_3$. Instead, Phases II and III each display a significantly extended χ distribution due to their hybrid nature. The distribution for Phase II is described by $\bar{\chi} = 1.428$ and $\sigma = 0.105$, which is skewed towards χ values approaching the pure Bloch value of $\pi/2$, but the existence of a competing Néel-type winding leads to a distribution of χ values clearly both less and broader than the pure Bloch case. This leads us to describe the hybrid skyrmions in Phase II as having a 'weak' Bloch character. On the other hand, the χ distribution for Phase III is described by $\bar{\chi} = 0.581$ and $\sigma = 0.223$, which describes a broader distribution that is skewed towards values below $\pi/4$, which is the mid-point between pure Néel and Bloch helicities. Therefore, we describe the hybrid skyrmions in Phase III as displaying a 'weak' Néel-type character.

Next, we next investigate the spatial distribution of helicity *within* the Néel, Bloch, Phase II and Phase III skyrmions. In the lower parts of main text Fig. 1a, b, e, and f, the variation of the colour in the schematics for Phases II and III already show the calculated helicity may vary with both radial distance from the skyrmion centre, and azimuthal angle within the plane of the two-dimensional skyrmion lattice. In Supplementary Fig. 8, we highlight this finding further by presenting representative one-dimensional (1D) cuts of the magnetisation texture that are

aligned with high-symmetry directions of the skyrmion lattices, and which traverse the skyrmion core.

Supplementary Figs. 8a and b show the real-space magnetisation distributions for model hexagonal Néel, and model hexagonal Bloch type skyrmion lattices, respectively. The lower parts of these figures show the same calculated 2D projections as shown in the lower parts of main text Fig. 1a and b. In both parts of Supplementary Figs. 8a and b, the same one-dimensional cut windows are indicated, and they are along both nearest neighbour (labelled i) and next-nearest neighbour (labelled ii) directions of the skyrmion lattice. Supplementary Figs. 8e and f respectively show both the magnetisation textures and calculated helicity along the 1D cut directions. As expected for model Néel and Bloch type skyrmion lattices, the helicity in the region of the cut is always either 0 or $\pi/2$ away from the centre (denoted by a white region at the centre of the strip), and thus remaining constant with both increasing radius from the core, and azimuthal angle within the skyrmion lattice plane.

Supplementary Fig. 8c shows the real-space magnetisation distribution expected for Phase II of $EuNiGe_3$. For this skyrmion type, spatial variations in helicity are apparent amongst the six different cuts labelled i to iv, which are presented in Supplementary Fig. 8g. While minor variations in helicity as a function of radial distance from the core can be discerned for cuts i to iii (which include the 3rd and 4th nearest neighbour directions), the helicity remains constant with radius for cuts iv to vi, which include the nearest and next-nearest neighbour directions. In contrast to the radial direction, it is clear that the skyrmion helicity displays a pronounced dependence on azimuthal angle in the skyrmion plane, with the strongest contrast between directions described by cuts i and iv.

Finally, Supplementary Fig. 8d shows the real-space magnetisation distribution expected for Phase III of $EuNiGe_3$, with Supplementary Fig. 8h showing the cuts along the next-nearest neighbour (cut i) and nearest neighbour (cut ii) skyrmion lattice directions. In this case the helicity along the next-nearest neighbour direction remains constant with radial distance from the skyrmion centre, and at a value larger than expected for the pure Néel skyrmion lattice. In contrast, along the nearest neighbour direction, the helicity varies strongly with radial distance, changing from a more Néel-like character near the core, to a Bloch-like character near the skyrmion edge. It follows that the Phase III skyrmions also further display a clear dependence of helicity on the azimuthal angle in the skyrmion plane.

Supplementary References

S1. Y. Fujishiro, N. Kanazawa, R. Kurihara, H. Ishizuka, T. Hori, F. S. Yasin, X. Yu, A. Tsukazaki, M. Ichikawa, M. Kawasaki, N. Nagaosa, M. Tokunaga, Y. Tokura, Giant anomalous Hall effect from spin-chirality scattering in a chiral magnet. *Nat. Commun.* **12**, 317 (2021).

S2. P. Schoenherr, J. Müller, L. Köhler, A. Rosch, N. Kanazawa, Y. Tokura, M. Garst, D. Meier, Topological domain walls in helimagnets. *Nat. Phys.* **14**, 465-468 (2018).

S3. H. Ishizuka, N. Nagaosa, Large anomalous Hall effect and spin Hall effect by spin-cluster scattering in the strong-coupling limit. *Phys. Rev. B* **103**, 235148 (2021).

S4. A. Neubauer, C. Pfleiderer, B. Binz, A. Rosch, R. Ritz, P. G. Niklowitz, P.Böni, Topological Hall Effect in the A Phase of MnSi. *Phys. Rev. Lett.* **102**, 186602 (2009).

S5. R. Ritz, M. Halder, C. Franz, A. Bauer, M. Wagner, R. Bamler, A. Rosch, C. Pfleiderer, Giant generic topological Hall resistivity of MnSi under pressure. *Phys. Rev. B* **87**, 134424 (2013).

S6. B. J. Chapman, M. G. Grossnickle, T. Wolf, M. Lee, Large enhancement of emergent magnetic fields in MnSi with impurities and pressure. *Phys. Rev. B* **88**, 214406 (2013).

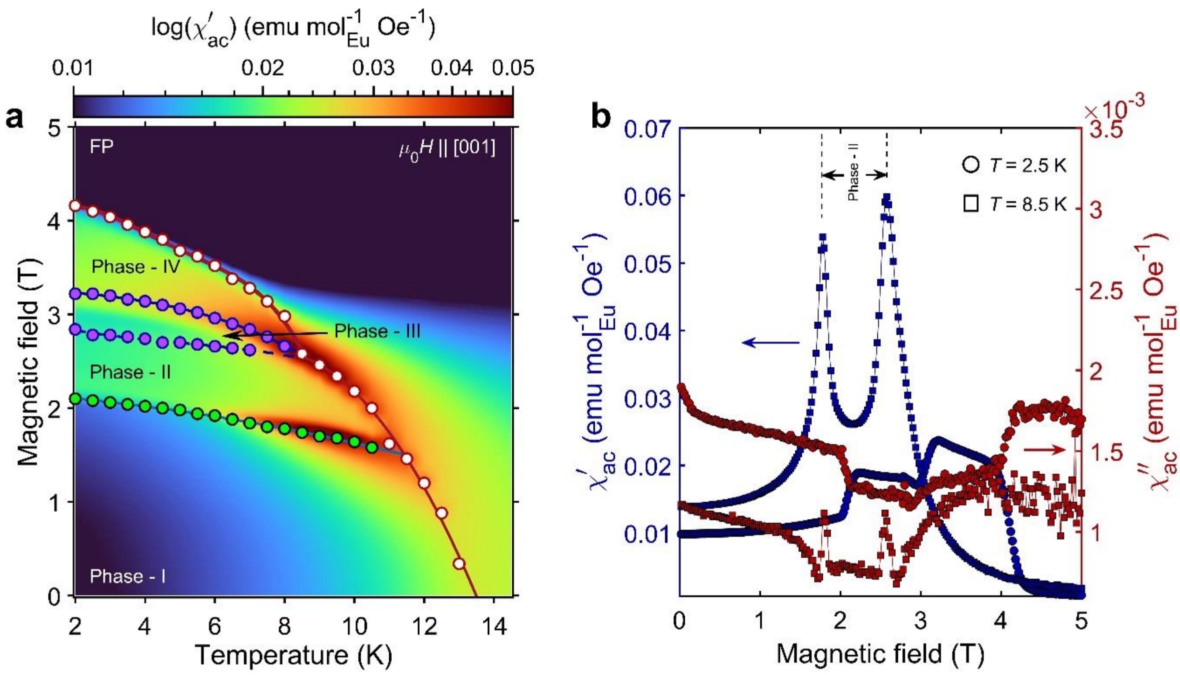

Supplementary Fig. 1 │ Phase diagram of EuNiGe$_3$ from ac susceptibility. a Magnetic phase diagram contour map of EuNiGe$_3$ constructed from of $\chi_{ac}'(H)$ data. In order to avoid hysteretic effects, the sample was demagnetised in the paramagnetic phase. As can be seen from the colour map, χ_{ac}' attains a lower value in Phase III compared to Phase II. **b** Representative isothermal *H*-scans of both χ_{ac}' and χ_{ac}'' measured at two different temperatures, 2.5 K and 8 K. The data reveal sharp phase transitions associated with both Phase II and III in the two contributing parts of the total susceptibility.

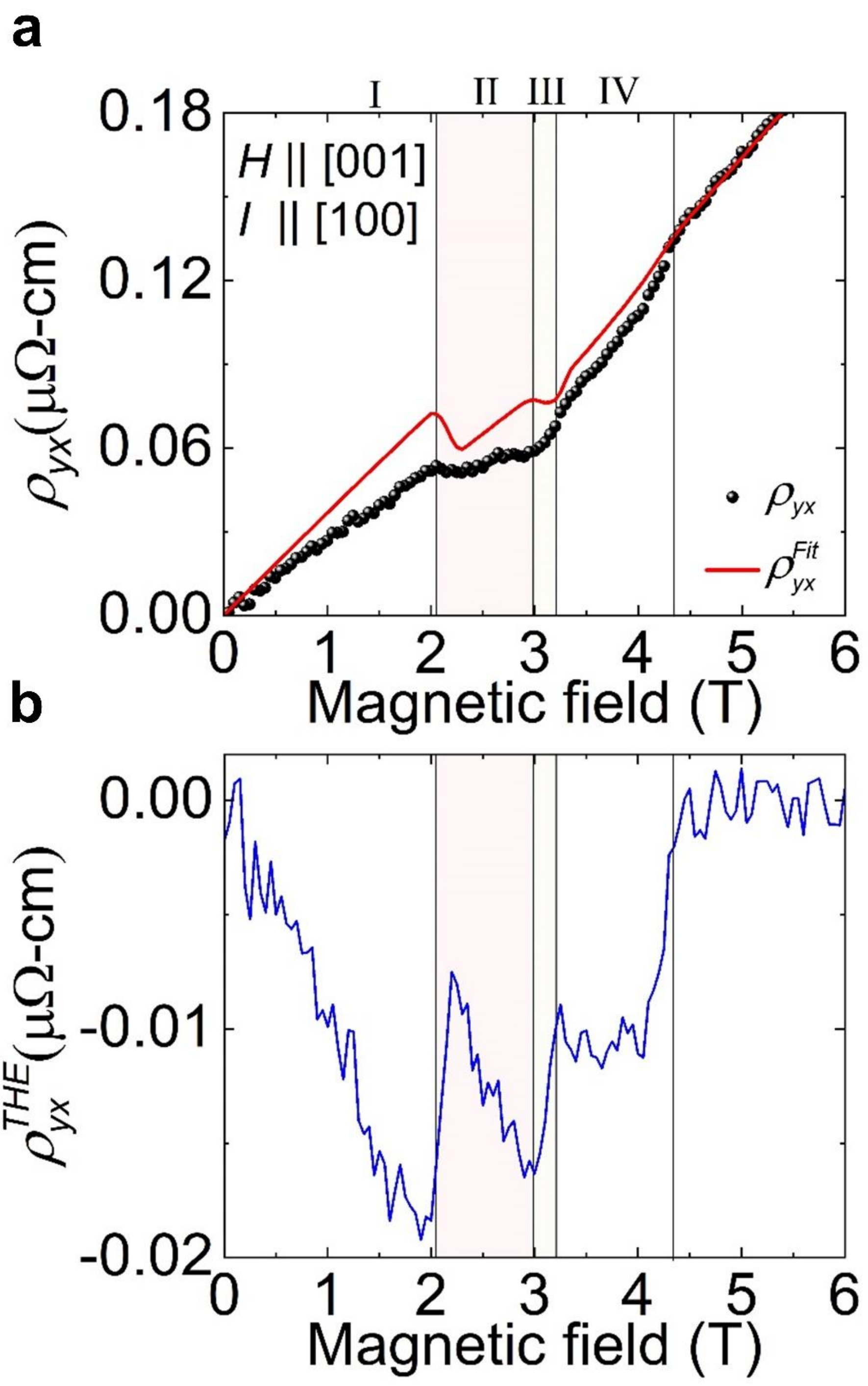

Supplementary Fig. 2 │ Topological Hall resistivity. a Field dependence of Hall resistivity, $\rho_{yx}(H)$, where the solid red line shows the fit by Equation 1 to the data of ρ_{yx}. **b** Topological contribution of Hall resistivity, ρ_{yx}^{THE}, for $EuNiGe_3$. Shaded pink and yellow regions respectively denote the field stability ranges of phases II and III.

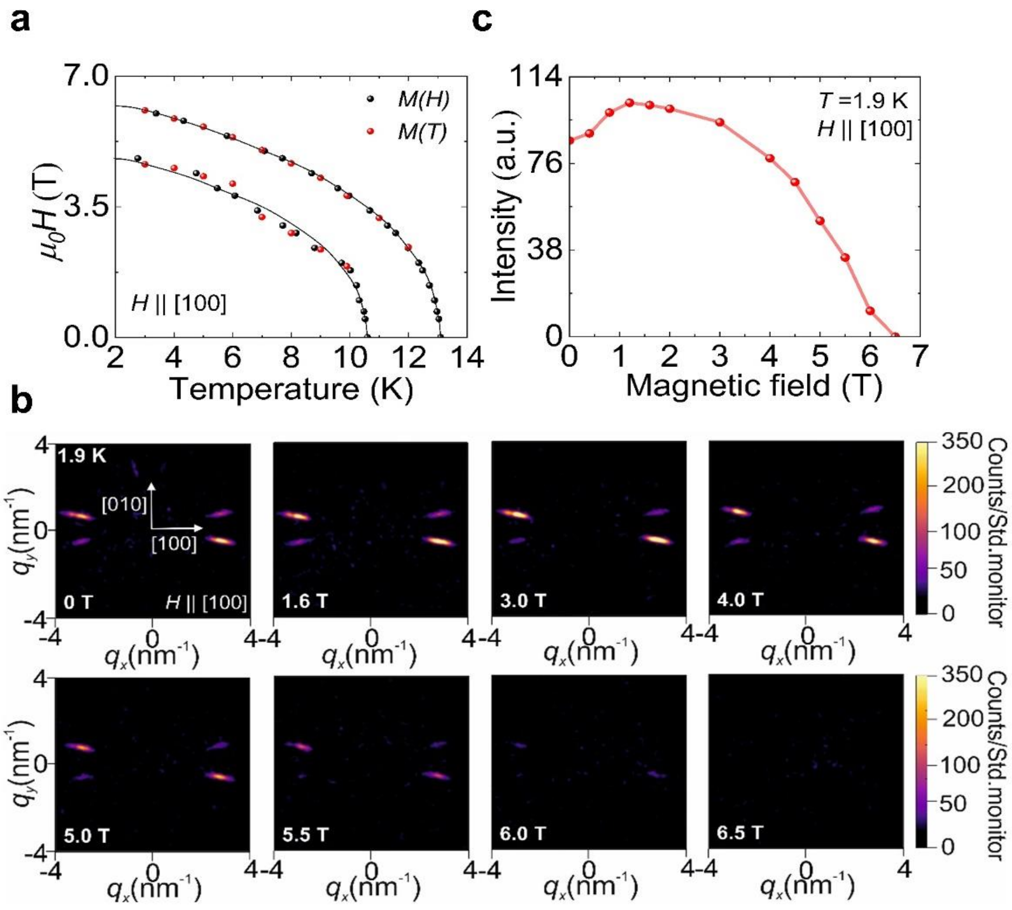

Supplementary Fig. 3 │ Bulk and SANS measurements for H || [100]. a Magnetic phase diagram for H || [100]. **b** SANS images measured at T = 1.9 K in the H || [100] geometry at selected fields. The colour scale indicates the scattering intensity. **c** The scattering intensity for the $\mathbf{Q_1}$ magnetic reflections as a function of magnetic field for H || [100]. In panel **c** error bars indicating the standard error are smaller than the symbol size.

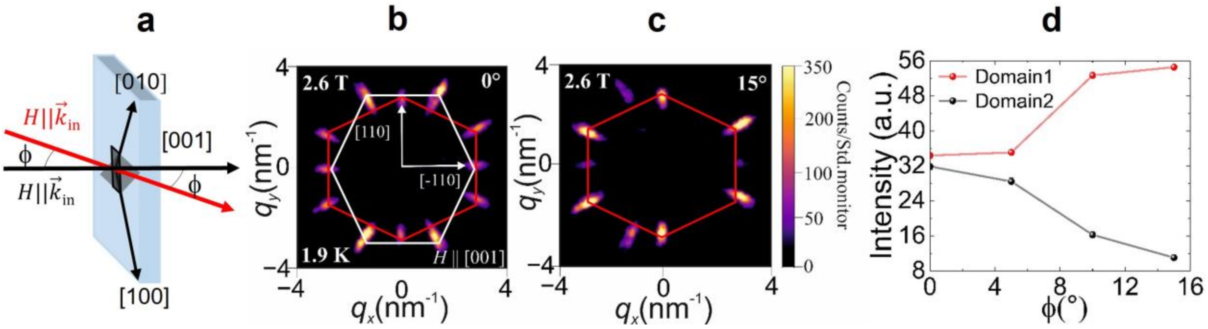

Supplementary Fig. 4 │ SANS data under tilted fields in Phase II. a Schematic side view of the experimental configuration. The field is tilted at different angles (ø) from the [001] direction in the (110) plane. **b** and **c** Representative SANS patterns obtained at $\mu_0 H$ = 2.6 T and T = 1.9 K, for ø = 0° (**b**) and 15° (**c**) from H || [001]. **d** The overall SANS intensity from the two triple-Q domains as a function of tilting angle ø. The data labelled as Domain 1 (Domain 2) correspond to those from the Bragg peaks marked by the red (white) hexagons in **b** and **c**. In panel **d** error bars indicate the standard error.

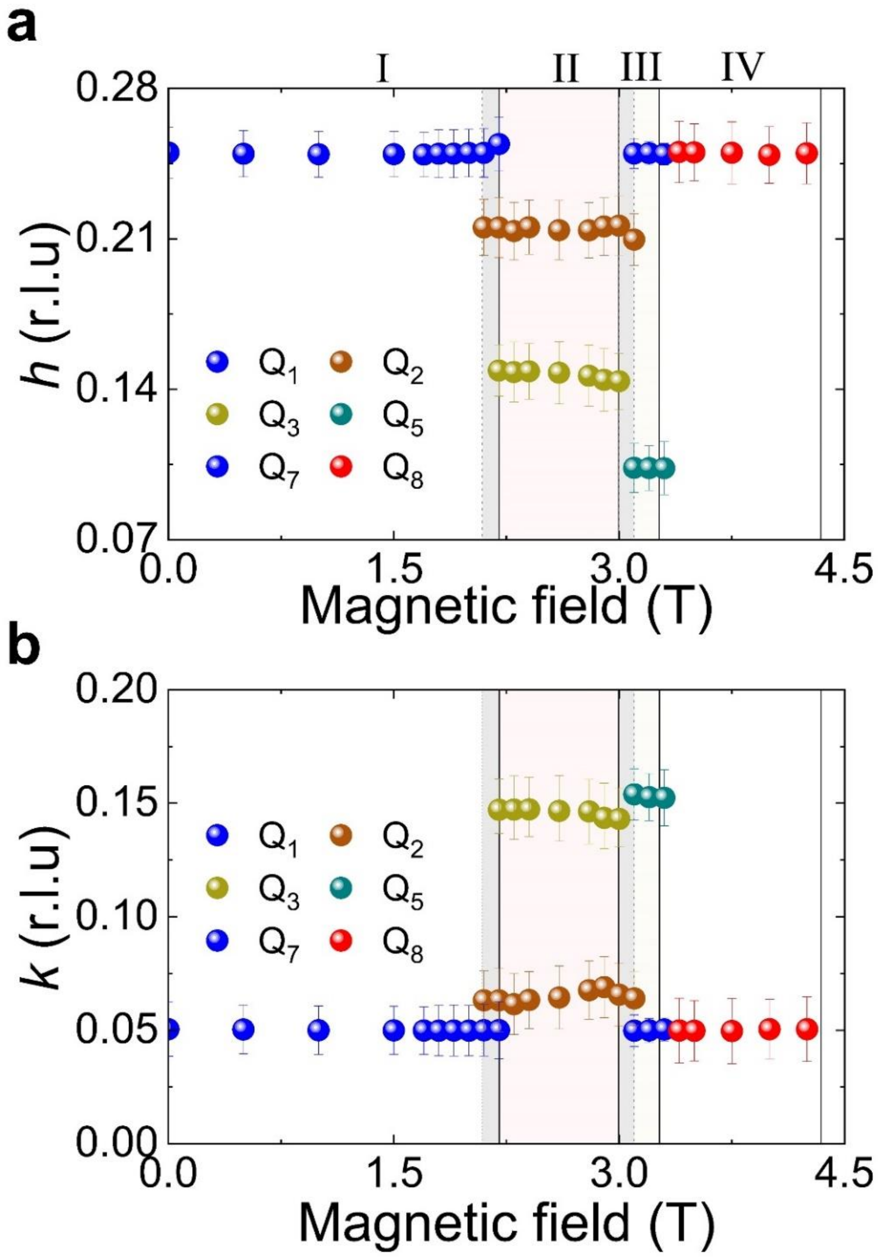

Supplementary Fig. 5 │ Field dependence of *h* and *k* components of magnetic Q-vectors. **a** and **b** The field dependence of the *h* (**a**) and *k* (**b**) values of the various symmetry-distinct *Q*-vectors in phases I to IV, as determined from the unpolarised SANS data. See Figs. 3i to l in the main text for definitions of the symmetry-distinct *Q*-vectors. In panels **a** and **b** error bars indicate the standard error. Shaded pink and yellow regions respectively denote the field stability ranges of phases II and III. The shaded grey regions denote field ranges of observed hysteresis.

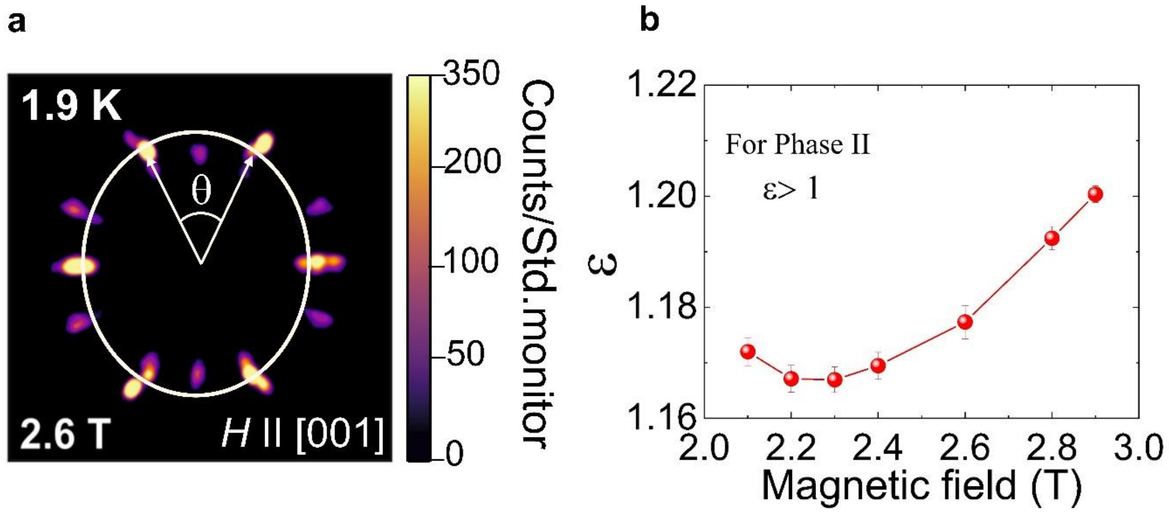

Supplementary Fig. 6 │ Parameterising the real-space skyrmion distortion from SANS data. a SANS data pattern obtained at 2.6 T and 1.9 K for H || [001]. The white ellipse overlays the SANS Bragg spots due one of the triple-Q domains. The angle θ describes the opening angle of the primitive cell of the 2D Bravais lattice that can describe the reciprocal lattice of the multi-Q structure. **b** The H-dependence of ε, which parameterises the axial ratio of the ellipse that overlays the Bragg spot distribution of the triple-Q domains in Phase II. This quantity provides a direct measure of the skyrmion distortion in real-space. In panel **b** error bars indicate the standard error.

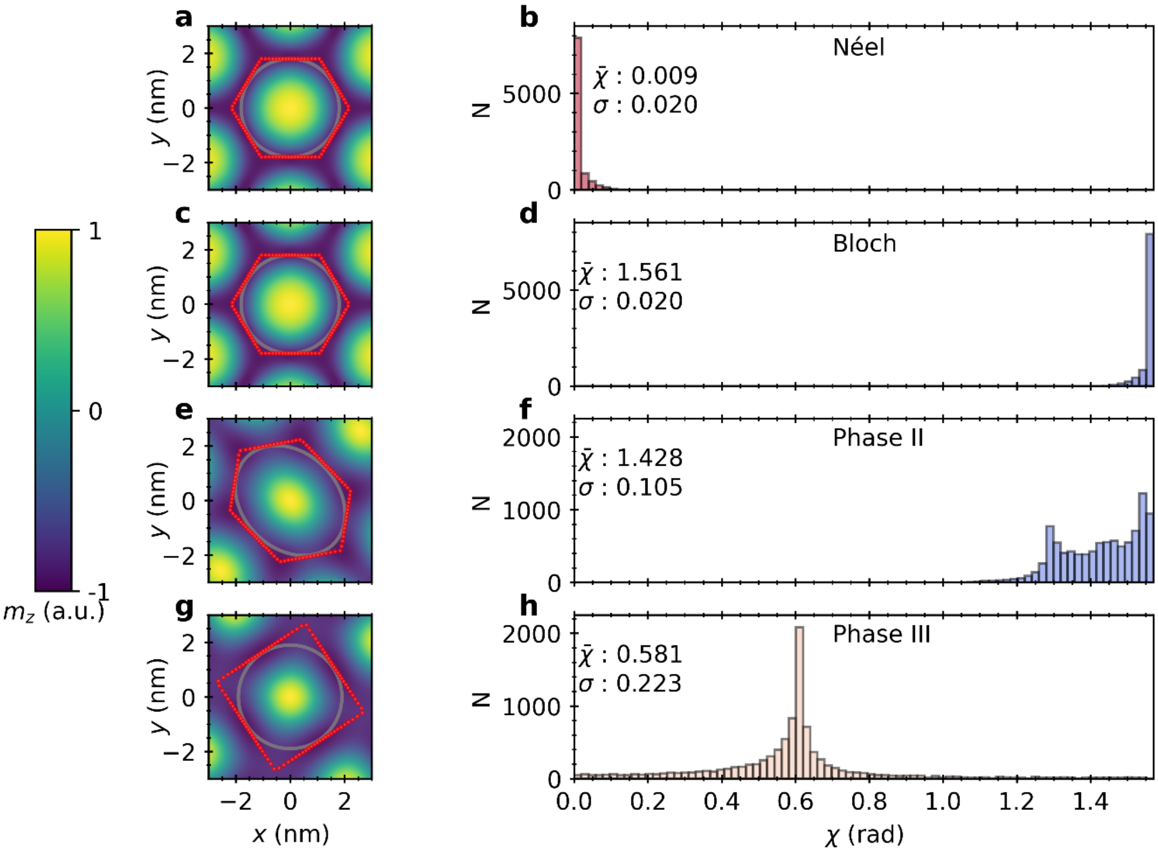

Supplementary Fig. 7 │ Distribution of helicity values (χ) for Bloch, Néel, and hybrid skyrmions. a Real-space representation of the two-dimensional magnetic unit cell for a model hexagonal Néel-type skyrmion lattice. The colour map encodes the m_z component. The red dots denote the perimeter of the magnetic unit cell, constructed from nearest-neighbour skyrmion bisections. The grey circle denotes the region characterized by an azimuthally symmetric skyrmion profile. **b** Histogram of calculated helicity (χ) values for the Néel skyrmion in the red region shown in **a**. Panels **c** and **d** report the same analysis for a model Bloch-type skyrmion lattice. Panels **e** and **f**, and panels **g** and **h**, respectively show the analysis for hybrid skyrmions in Phase II and Phase III of $EuNiGe_3$. In all panels **b**, **d**, **f**, and **h**, the helicity bin width is 0.0196 units of χ. The insets provide both the mean helicity $\bar{\chi}$ and a measure of the standard deviation σ, of the helicity distribution.

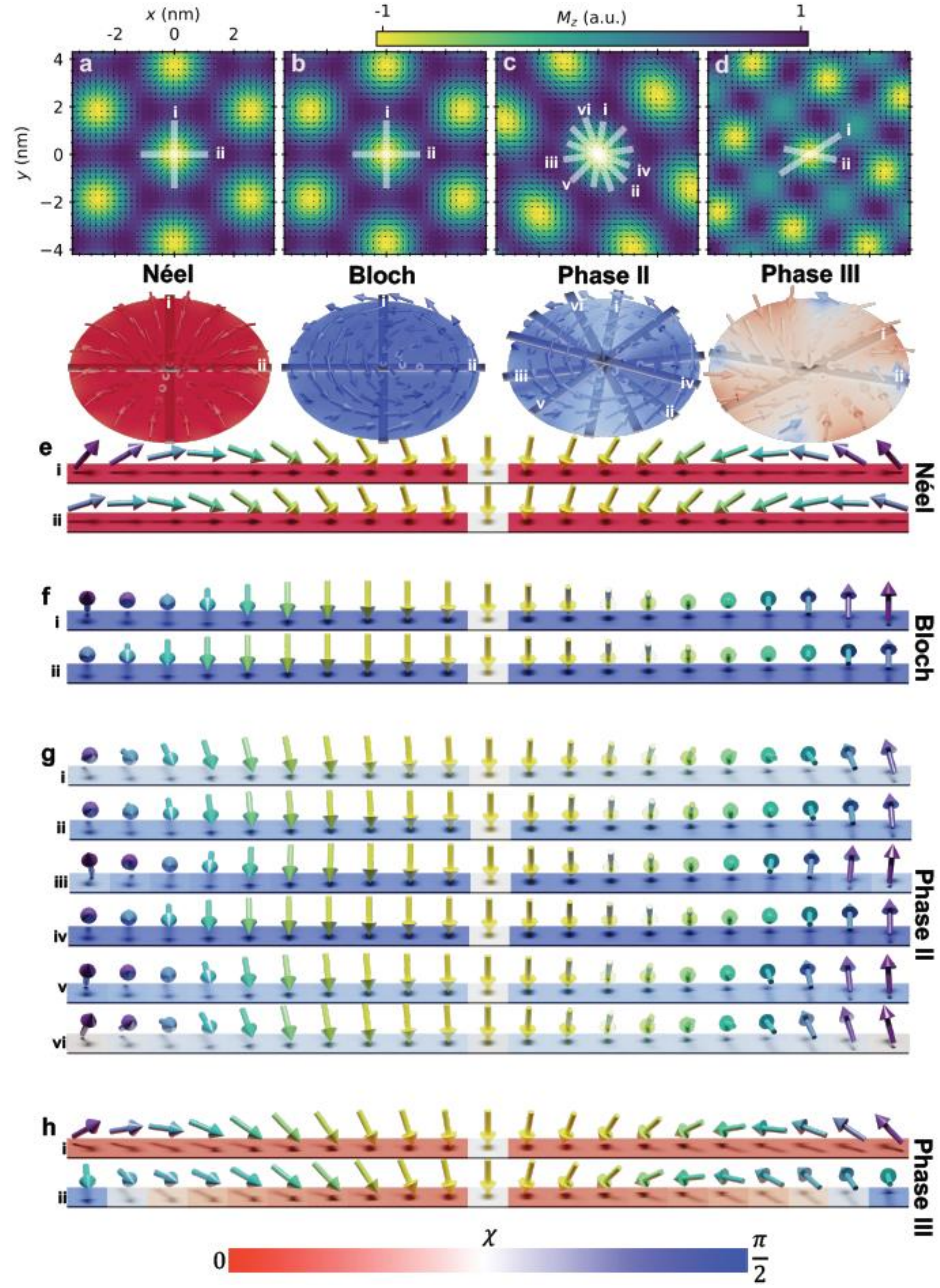

Supplementary Fig. 8 | Spatial distribution of helicity for Bloch, Néel, and hybrid skyrmions. Real-space representations of **a** a hexagonal Néel-type skyrmion lattice, **b** a

hexagonal Bloch-type skyrmion lattice, **c** Phase II of $EuNiGe_3$, and **d** Phase III of $EuNiGe_3$ are shown in the upper parts of each panel. The colour map encodes the m_z component, while arrows indicate the direction of in-plane magnetisation. The white bars with roman numerals indicate cuts along high symmetry directions of the skyrmion lattice, and over which the spatial dependence of the helicity is calculated as shown in panels in **e** to **h**. The lower parts of panels **a** to **d** show the relevant 2D skyrmion projections originally presented in Figs. 1a, b, e and f, also overlaid with the same cut bars as shown in the upper panels. The colour of the discs encodes the spatial distribution of the calculated helicity χ, and in accord with the relevant colourbar at the bottom of the figure. **e** The spatial dependence of the calculated helicity along the nearest-neighbour (cut i) and next-nearest neighbour direction (cut ii) of the Néel-type skyrmion lattice. The arrows indicate the local direction of the magnetisation, and they are coloured according to their m_z component (the colourbar above panels **a** to **d** applies). The colour of the strip underneath the arrows encodes the spatial dependence of calculated helicity χ, according to the colourbar at the bottom of the figure. **f** The same as for **e** but for a model Bloch type skyrmion lattice. **g** and **h** respectively show the cuts along the high symmetry skyrmion lattice directions for Phases II and III of $EuNiGe_3$.
